# Supplementary figures and images for: The confounding effects of eye blinking on pupillometry, and their remedy
Source: PLoS One. 2021 Dec 17;16(12):e0261463. doi: 10.1371/journal.pone.0261463 (PMC8683032; doi:10.1371/journal.pone.0261463)

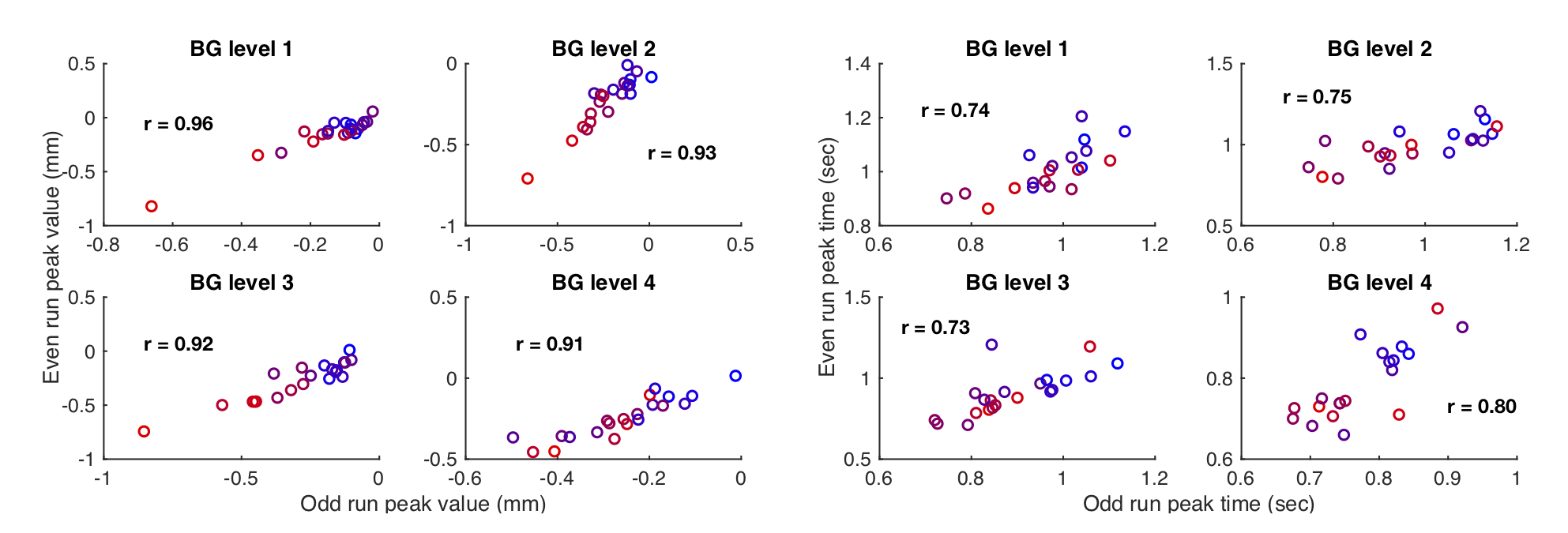

Supplement: S1 Fig — (left panel) Split-half correlation in peak amplitudes (right panel) Split-half correlation in peak time. (TIFF) [file pone.0261463.s001.tiff]

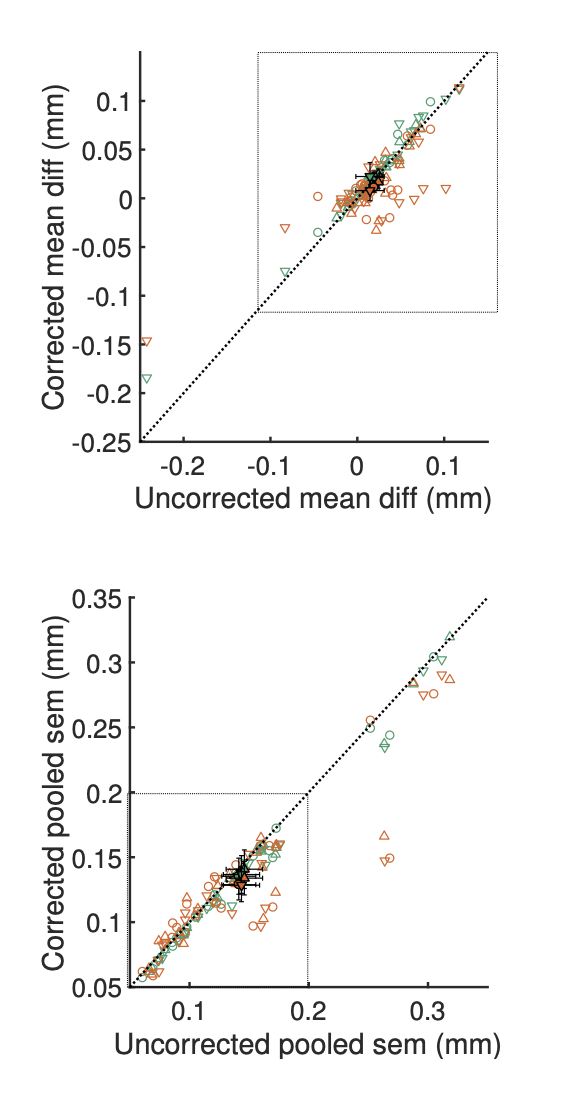

Supplement: S2 Fig — (TIFF) [file pone.0261463.s002.tiff]
