# Supplementary material for: The confounding effects of eye blinking on pupillometry, and their remedy
Source: PLoS One. 2021 Dec 17;16(12):e0261463. doi: 10.1371/journal.pone.0261463 (PMC8683032; doi:10.1371/journal.pone.0261463)
Supplement: Attachment — Submitted filename: review.pdf [file pone.0261463.s004.pdf]

Review

Yoo, Ahn, & Lee (2021) The confounding effects of eye blinking on pupillometry, and a remedy for them. *PLoS ONE.*

In this article, the authors describe the blink-induced pupil response (BPR), which is a pronounced pupil constriction from about 200 to 3000 ms after a blink. They point out that this not only increases noise when measuring pupil size, but is also likely to introduce confounds when blink rate covaries with the experimental conditions (as it often does). They then outline a technique to remove the PBR from the pupil time series, and show that this increases statistical power.

I feel that this is a very well-done study. It's well-written, despite some sentence-level English mistakes (but the structure is well done). And the aim and conclusions are clear and reasonable. I have a few suggestions to further strengthen the manuscript.

The introduction does not address the question of *why* the pupil constricts after blinks. In a sense, this is not important, because in the context of the current manuscript it's mostly relevant that it does (and not necessarily why). However, I suspect most readers will still be left wondering, and so I would add a short paragraph about this. I know this probably comes across as a cheap excuse to ask for a citation (I don't mean it as such), but I speculated on this in a recent review article, in which I basically suggest that it's the same phenomenon as the pupil constriction that is induced by visual change:

- https://www.annualreviews.org/doi/full/10.1146/annurev-vision-030320-062352

More importantly, I feel that the manuscript would benefit from practical guidelines for researchers to actually apply this method. It seems that the OSF repository includes code to conduct the analyses, or at least there's a `codes.zip` file. But this file seems to be corrupted, and in any case the organization of the OSF and to what extent it contains useful code is unclear. So cleaning up and documenting the OSF project would be low-hanging fruit.

And what kind of practical considerations should researchers keep in mind? For example, when using an EyeLink, you generally start recording at the start of a trial, and then end recording at the end of the trial. This means that there's no continuous pupil time series that spans multiple trials. To what extent is this a problem for applying the proposed technique? More generally, a paragraph with some hands-on advice would be worthwhile, in my opinion.

Sebastiaan Mathôt
Department of Experiment Psychology

University of Groningen
http://www.cogsci.nl/smathot
