# Supplementary material for: The confounding effects of eye blinking on pupillometry, and their remedy
Source: PLoS One. 2021 Dec 17;16(12):e0261463. doi: 10.1371/journal.pone.0261463 (PMC8683032; doi:10.1371/journal.pone.0261463)
Supplement: Attachment — Submitted filename: PONE-D-21-12636.pdf [file pone.0261463.s005.pdf]

Review

Kyung Yoo, Jeongyeol Ahn, & Sang-Hun Lee1 (2021). The Confounding Effects of Eye Blinking on Pupillometry, and a Remedy for Them. *PlosOne***.**

In the present study the authors explain the BPR problem, the possible artifact that it might cause and also an algorithm to solve it. While the main idea is important and should be discussed and solved, I found three main issues that should be addressed before publication.

1. The authors present and analyze the pupil size that was changed due to the BPR. However, it's not clear whether eye blinks were caused / associated with the observed pattern. For example, in Fig. 5C it seems that there are dips after about 5 seconds and 20 seconds. These dips looks pretty much the same as other dips that were associated by the authors to eye blinks and therefore to BPR. Moreover, it seems that there are oscillations in pupil size that cause dipping about every 5 seconds (0.2 Hz). There are several possible options to solve this problematic issue. One possible options is to compare two groups – one group that will be allowed to blink, and another group that won't be able to blink (by using dedicated eye drops). Another possible option is by comparison between trials / time windows with blinks and those without blinks. If the authors find meaningful differences between the groups, the interpretation of the original results will be more reliable. Without this comparison it is difficult to understand the weight of this possible artifact in pupillometry studies.
2. In addition to the (relatively) steady state measurement of the pupils (the fixation task), the authors ran more two independent cognitive tasks. While the results of the fixation task were relatively replicated in these task, the main idea behind the possible artifact is still not clear. Yes, indeed, the mean was pretty much the same and the scattering was decreased (this makes sense with respect to the first main issue that caused interpolation of a lot of dips). However, the influence of this possible artifact on the actual effects unclear. From the study perspective, the BPR might cause systematic pupil decrement that is not associated with the task (this is a bit of a

problematic statement due to the fact that the authors cited studies that associated eye blink rate with task difficulty), therefore, effects should appear (false positive) / disappear (false negative) after the BPR correction. If this is the case here, the authors should present these possible scenarios with / without BPR corrections. Specifically, on one hand, the authors should show effects (in terms of the actual task – e.g., easy vs. difficult) that will appear after correction, and on the other hand, the authors should show effects that will disappear after correction. Without these comparisons, the contribution of the correction is not clear (at least in cognitive tasks).

3. The authors present a model that should solve the BPR artifact. While the model without any doubt changed the general pattern of the pupil size, it is not clear if it was done correctly. Specifically, it is not clear whether the pattern of the pupils after the correction is in line with the expected pattern of the pupils in the case of no eye blinks and moreover, without PBR. Without this important comparison, it is impossible to evaluate the efficiency of the authors' algorithm.

In addition to these three major issues, there are several (relatively) minor issues:

1. Lines 138-142: I'm not entirely sure what is happening here.
2. In most of the figures the legends are missing.
3. Something doesn't make sense in Fig. 7. After 4 seconds there are a lot blinks (the EBR is relatively large), but the pupil size is still increased (with / without corrections). How it is in line with the main idea of BPR? Why the pupil is sometimes increased and sometimes decreased?
4. Lines 569-574: Was the exclusion (of both trials and subjects) also based on task performance? If not, why?
5. Lines 603-609: Why were the fixation colors different between trials? With respect to the first major point, maybe these contrasts were the reason for the 0.2Hz oscillations.
6. Line 653: How did the authors separate between eye-blinks and other artefacts (e.g., noise / head movements).
7. Lines 653-656: Please note that this approach is less efficient compared to the noise-based approach (Hershman et al., 2018).

8. Line 659 ("Blink onsets and offsets were defined from the start and end points of the blinks"): This sentence is not clear. How were the onset and the offset defined?
9. Lines 663-665: A period of 150 ms is problematic. Please see Hershman et al.'s (2018) discussion about it.
10. Lines 667-672: This part is not clear. Why was smoothing required?
11. Lines 773-776: Why were these time windows chosen?
12. The BPR correction algorithm (lines 722-768): The algorithm is pretty difficult to follow. Simplification of this section is required. In addition, did the authors share the source code? Free downloading of the Matlab / Python / R code would be useful here (both for the readers and for the reviewers).

Ronen Hershman.

Department of Cognitive Neuroscience,

Ben-Gurion University of the Negev, Beer Sheva, Israel.

https://in.bgu.ac.il/en/Labs/CNL/Pages/staff/ronenhe.aspx
