# Supplementary material for: The confounding effects of eye blinking on pupillometry, and their remedy
Source: PLoS One. 2021 Dec 17;16(12):e0261463. doi: 10.1371/journal.pone.0261463 (PMC8683032; doi:10.1371/journal.pone.0261463)
Supplement: Attachment — Submitted filename: review_PONE-D-21-12636R1.pdf [file pone.0261463.s007.pdf]

Review

Kyung Yoo, Jeongyeol Ahn, & Sang-Hun Lee1 (2021). The Confounding Effects of Eye Blinking on Pupillometry, and a Remedy for Them – R1. *PlosOne***.**

I have read the authors' responses and the revised manuscript and find that most of my concerns are still there. Moreover, it made me wonder about additional issues that I didn't notice in the first version.

As I mentioned in my previous review, while the main idea is important and should be discussed and solved, there are several issues that should be addressed before publication should be considered. The most important issue is the validity of the algorithm. Specifically, the authors presented partial analysis (i.e., only for two experiments) that estimated the efficiency of their algorithm. Another important issue is about the way they estimated the efficiency of their algorithm; namely, what they compared (or didn't compare). In my opinion, there are difficulties that make the conclusion unclear. The third main issue (and maybe the most serious one) is about the methodology that the authors used in their studies. Specifically, it seems that the second experiment had short durations that cannot be used in pupillometry studies (due to lack of time to get back to baseline). In addition, it seems there are several methodological issues in their analysis; specifically, the smoothing that might destroy valid data, comparison that cannot provide information about the influence of the manipulation on the pupil size, and possibly wrong blink corrections. These are all a little bit problematic in a study that aims to solve a methodological issue. Another important issue is that the authors didn't provide any real indication of the importance of the BPR correction. Yes, indeed, statistical power is highly important, but without a clear-cut example for the problematic uncorrected BPR, I can't see any reason to adopt the authors' algorithm. In other words, if it is impossible to present an example that will show how the correction helps researchers, there is no actual reason to use it.

As I mentioned above, I read the author's comments and the revised manuscript and would now like to refer to specific difficulties that I found in their responses and in the manuscript. First, I would like to address the authors' responses to the main comments (the identification of the comment is what the authors used in their response).

[C1-2]: I don't really understand the rationale behind the authors' test. In each trial there were two options: there was a correction of BPR (by using the authors' algorithm) or there was no correction. Why did the authors choose a different separation of trials? Moreover, in my opinion the analysis should be applied to all the experiments (to support the existence of the problem) and not only to the first two experiments. In addition, the comparisons have to be identical across experiments in terms of separation criteria. Actually, the presented analysis (see more comments about the presented analysis below) does not answer the important question: Does the authors' algorithm solve the problem?

I think I wasn't clear in my previous review so I'll try to explain it now. In Figure 4B (the one with a time window of 35 seconds), there is a decrement of the pupils about every 5 seconds = 0.2 Hz. Hz means occurrences per **second**. 2Hz means 2Hz occurrences per second. Here there is less than one occurrence per second. Hence, the frequency has to be smaller than 1. In Figure 4B there is one occurrence every 5 seconds, namely 1/5s = 0.2Hz. These decrements (or oscillations in the terminology of the original comment) are very strange and made me think maybe it might explain part of the results of the authors. These decrements (as far as reflected in the figure) are not associated with eye blinks (because their number is larger than the number of the observed eye blinks). If this is the case, I'm not entirely sure what the meaning of the analysis is and moreover, what the validity of the present study is.

[C2-2]: If I understand correctly, the authors said that the BPR might not cause a statistical problem (in terms of type I / type 2 errors) but could cause a decrease the statistical power. Moreover, in a series of three experiments, they failed to show why the correction (that it is not clear to me whether it is valid or not) is good and important. Yes, indeed, statistical power is important and is the

basis of any statistical test. However, in the presented work, I can't see any reason to use it. This is because the problem (i.e., in what scenarios the BPR will cause trouble) is not clear or well defined, and because the correction wasn't compared (convincingly) with different valid tools / cases without BPR correction.

[M2-3]: The authors' response is a bit problematic. If there are no decrements after any blink, why do the authors associate the decrements after blinks to blinks – this is the main idea of the study (unless I missed something). If the aim is to define the BPR (in terms of shape and latency), this issue should be of concern. Otherwise, it means that in several cases (that cannot be predicted – at least by me) the algorithm might destroy valid data.

[M2-4]: Performances should be added to support the authors' claim.

[M2-6]: Every device has noise. It means that any device might show extreme pupillometry values by mistake. My recommendation is to exclude extreme values by using Z-scores (in most cases 2.5 Z-scores) based on the mean and the SD of each trial separately. Please note that eye-movements can also cause missing values that might be associated accidently to eye-blinks (e.g., if the participant looks at the floor). In addition, if the participant touches his/her face (it might happen sometimes), missing values will be observed. Other scenarios that I can think about are associated with moving the head back. Yes, indeed, the chinrest is supposed to decrease the possibility of head movements, but it might happen. I highly recommend the authors check this, specifically in methodological work like this.

Beside my comments to the authors' answers, I have a (relatively long) list of specific comments (the numbers represent the relevant line in the manuscript).

30-38: "This pupil-size dynamics, which is **dissociated** with light": this is not absolutely true. Steinhauer found an interaction between them (Steinhauer, S. R., Siegle, G. J., Condray, R., & Pless, M.

(2004). Sympathetic and parasympathetic innervation of pupillary dilation during sustained processing. *International Journal of Psychophysiology, 52*(1), 77-86).

152-155: I looked for studies about the "delayed orientation estimation" and pupillometry, and I didn't find anything relevant. Moreover, the citation (44) is not relevant to this task. Hence, I'm not entirely sure why this task was chosen nor why the authors declared that "*These two tasks were chosen because the cognitive processes underlying task performance are relatively well established, and* ***those processes are known to be tightly associated with pupillary responses***".

156-173: I took a look at the cited paper and the authors in that paper made a different analysis (depending on the luminance). It is difficult to understand whether the results of the authors of the present manuscript are good or not in terms of replication.

193-204: Again, it is difficult to understand if the results are good or not due to the fact that they weren't compared with previous results in terms of replication.

353-379: Something is a bit unclear in Fig 6A. It seems that the pupil size in the trials with BPR and without corrections was larger than in trials when no corrections were required. I expected to see a huge dip (due to the BPR) that would be fixed after the presented correction. However, it seems that during these trials, the pupils had no decrements that associated with BPR. If I understand correctly, this figure suggests that only one peak was found during these trials. It seems to me that something went wrong with the analysis of those trials. In addition, I'm wondering why this analysis wasn't done on the third experiment also? In my opinion, the same test is supposed to be done on all the three experiments.

454-466: There were differences between the conditions **at time 0**. Therefore, it is difficult to conclude that the observed differences **after** the manipulation (i.e., the presentation of the stimulus) were caused by the differences themselves. In my opinion, the authors should present relative changes (compared to the stimulus onset, for example?). Moreover, at the trial **offset** (the beginning of trial n+1)

the pupil size was not equal to that at the trial **onset** (the beginning of trial n). Due to the fact that trial n and trial n+1 are identical in terms of the presentation of the mean response, it is problematic that the trial onset wasn't identical to the trial offset. It means that there was an influence of trial n on trial n+1 (in addition to the theoretical influence of the BPR). In other words, there was a methodological issue in the experiment. A study that deals with (relatively minor?) artifacts like BPR cannot use an experiment with a critical problem like that.

504-522: The summary talks about the algorithm itself that wasn't described at all in the manuscript. What is the purpose of the study? To describe the BPR issue? To address the BPR issue? Up until this part in the manuscript, no details about the algorithm were provided. How are the readers supposed to judge the algorithm?

610-618: The authors said that the other approach is not good enough because of the two mentioned crucial aspects. However, no comparison between the approaches was applied. If the authors suggest that their approach (that wasn't discussed in the manuscript except for in the last section) is better, a comparison should be done. In my opinion, a comparison between the 2 approaches and between the data after the BPR corrections to trials with no BPR in the three experiments has to be done.

625-627: This is a serious problem. It means that the algorithm might destroy valid data with a wrong interpolation. Isn't it safer to ignore the BPR?

641-646: In my opinion, the "back to baseline" is a good reason for a relatively long ITI. As it is reflected in Exp. 2, this might also be problematic if there are no blinks (and BPR) at all.

647-652: Please note that this is also really relevant in case of blink corrections. Actually, in general it is highly recommended to measure pupil size before the manipulation onset. As reflected from Fig. 9, there are differences in the manipulation onset. Actually, we don't really know what was happening before the manipulation onset in terms of the pupil size. Therefore, it is difficult to conclude

that the manipulation itself caused the results we see. Specifically, in addition to the comparison relative to the stimulus onset, the researcher has to be sure that any changes in the pupil size didn't start **before** the manipulation itself. Hence, the recommendation about recording the data before the manipulation onset is not **only** relevant for BPR, but for pupillometry studies in general (also for studies when there are no eye blink at all by using special drags …).

653-663: In contrast to RT studies (but not only), in pupillometry studies it is important to get data from the eyes. Hence, **voluntary** eye blinks (for a rest?) are not highly recommended. Honestly, I don't believe that it is possible to avoid involuntary eye blinks (that are associated with all the aspects that the authors mentioned). Hence, I think this suggestion (avoid researchers to ask participants to avoid eye blinks) is wrong and might cause loss of data.

701-702: The authors decided to not give the subjects any instructions about eye blinking. They also recommended other researchers do the same (as reflected from lines **653-663**). Hence, it seems that there is a conflict of interest in their recommendation, specifically if they didn't examine it directly; namely, if they did not compare between two groups that are different based on the instructions.

763-766: Did the authors find meaningful differences between the two eyes? If the authors decided to use binocular measurement, why wasn't the mean pupil size (of the two eyes) used for the analysis?

785-787: In my previous review I asked why the smoothing was required. I understand that the authors asked to reduce the noise of the data, however, smoothing with a boxcar time window of 250 ms not only reduces the noise, but also the signal itself. Actually, a boxcar time window of 10 ms should remove most of the noise. Therefore, it seems to me that the smoothing was too aggressive and in my opinion should be a new concern. EyeLink devices are well known to be high quality devices. Hence, smoothing is not really required in pupillometry studies. For reference, in our lab we are using the eye tribe devices (that have a sampling rate of 60 Hz and are well known to be relatively noisy devices). After

blink correction, no smoothing is required. The reason that I mention it again (after I asked about it in my previous review) is due to the data in Figure 6. In this figure, the data looks too processed and it made me question whether the smoothing might have caused these patterns.

794-795: If the reason behind the smoothing is to remove noise, why was the band-pass filter also required? Was the smoothing used only for the detection of the blinks? It is not clear to me.

817-826: Similar to the general comment about the separation between trials, I don't really understand this rationale. In each trial there were two options—there was a correction of BPR (by using the authors' algorithm) or there was no correction. Why do the authors separate the trials differently?

Ronen Hershman.

Department of Cognitive Neuroscience,

Ben-Gurion University of the Negev, Beer Sheva, Israel.

https://in.bgu.ac.il/en/Labs/CNL/Pages/staff/ronenhe.aspx
