# Supplementary material for: The confounding effects of eye blinking on pupillometry, and their remedy
Source: PLoS One. 2021 Dec 17;16(12):e0261463. doi: 10.1371/journal.pone.0261463 (PMC8683032; doi:10.1371/journal.pone.0261463)
Supplement: Attachment — Submitted filename: Response to Reviewers.docx [file pone.0261463.s006.docx]

Point-to-point replies to the reviewers’ comments

**Major comments: [C1-1] ~ [C3-3]**

**[C1-1]** Reviewer #1’s overall comments on our manuscript

*“In this article, the authors describe the blink-induced pupil response (BPR), which is a pronounced pupil constriction from about 200 to 3000 ms after a blink. They point out that this not only increases noise when measuring pupil size, but is also likely to introduce confounds when blink rate covaries with the experimental conditions (as it often does). They then outline a technique to remove the PBR from the pupil time series, and show that this increases statistical power.”*

*“I feel that this is a very well-done study. It's well-written, despite some sentence-level English mistakes (but the structure is well done). And the aim and conclusions are clear and reasonable. I have a few suggestions to further strengthen the manuscript.”*

**Our reply to [C1-1]**

We thank the reviewer for pointing out the key findings and contribution of our manuscript. We’re also thankful for the reviewer’s nice comments on the overall quality of our work. On the other hand, we admit (and regret) that we did not spend sufficient time and effort on making our manuscript readable and sound fluent. In the revised manuscript, we did our best to correct the entire manuscript for grammatical errors and tried to improve its readability and fluency by incorporating the comments and suggestions from writing experts.

**[C1-2]** Reviewer #1’s comments on “why BPR occurs”

*“The introduction does not address the question of *why* the pupil constricts after blinks. In a sense, this is not important, because in the context of the current manuscript it's mostly relevant that it does (and not necessarily why). However, I suspect most readers will still be left wondering, and so I would add a short paragraph about this. I know this probably comes across as a cheap excuse to ask for a citation (I don't mean it as such), but I speculated on this in a recent review article, in which I basically suggest that it's the same phenomenon as the pupil constriction that is induced by visual change:.”*

**Our reply to [C1-2]**

We concur with the reviewer that readers might wonder or be curious about “why the pupil constricts after blinks” although this issue is beyond the current work scope. As the reviewer suggested, we addressed this issue by writing a new sentence in INTRODUCTION and adding a new section, titled ‘Potential causes of BPR’, in DISCUSSION. In INTRODUCTION (right after introducing BPR, lines 42 ~ 44), we just briefly mentioned the ‘visual change’ as the most likely cause for BPR with a couple of key references, to ease readers’ curious mind about BPR. In DISCUSSION (‘Potential causes of BPR’, lines 524 ~ 537), we further detailed the potential causes of BPR with more references for the readers who want to be informed of the previous work on the matter.

**[C1-3]** Reviewer #1’s comments on practical guidelines for using the correction algorithm for BPR correction

*“More importantly, I feel that the manuscript would benefit from practical guidelines for researchers to actually apply this method. It seems that the OSF repository includes code to conduct the analyses, or at least there's a `codes.zip` file. But this file seems to be corrupted, and in any case the organization of the OSF and to what extent it contains useful code is unclear. So cleaning up and documenting the OSF project would be low-hanging fruit.”*

**Our reply to [C1-3]**

First of all, we feel sorry for the corrupted file provided at the OSF repository at the time of submission of the previous version of the manuscript. We’ve re-uploaded the file at the OSF repository (<https://osf.io/uvs2d/>) and checked whether it is downloadable and work smoothly. In addition, to help users apply our BPR-correction method to their own data with ease, we reorganized the codes in the toolbox, provided a document that explains the key steps for BPR correction including data preprocessing, and demonstrated those procedures with an example data set (https://github.com/yookyung1310/BPR_toolbox). In the revised manuscript, we added a paragraph in the DISCUSSION section of “The proper way of correcting pupil-size measurements for BPR” and added a sentence at the end of METHODS and MATERIALS, where we instructed readers how to access and use our BPR-correction toolbox.

**[C1-4]** Reviewer #1’s comments on hands-on advice for pupillometry researchers

*“And what kind of practical considerations should researchers keep in mind? For example, when using an EyeLink, you generally start recording at the start of a trial, and then end recording at the end of the trial. This means that there's no continuous pupil time series that spans multiple trials. To what extent is this a problem for applying the proposed technique? More generally, a paragraph with some hands-on advice would be worthwhile, in my opinion.”*

**Our reply to [C1-4]**

We thank the reviewer for directing our attention to this very important point. We fully recognize the importance of offering such hands-on advice to readers, who are potential users of pupil data, based on what we found in the current work. So, in the revised manuscript, we added a new section titled “Practical considerations for avoiding BPR-related problems” in DISCUSSION. In this newly added paragraph, we listed to-dos and not-to-dos over various stages of experimental design to data preprocessing.

**[C2-1]** Reviewer #2’s comments on the association between eye blinks and the observed patterns of pupil size

*“In the present study the authors explain the BPR problem, the possible artifact that it might cause and also an algorithm to solve it. While the main idea is important and should be discussed and solved, I found three main issues that should be addressed before publication.*

*1. The authors present and analyze the pupil size that was changed due to the BPR. However, it’s*

*not clear whether eye blinks were caused / associated with the observed pattern. For example, in*

*Fig. 5C it seems that there are dips after about 5 seconds and 20 seconds. These dips looks pretty*

*much the same as other dips that were associated by the authors to eye blinks and therefore to*

*BPR. Moreover, it seems that there are oscillations in pupil size that cause dipping about every 5*

*seconds (0.2 Hz). There are several possible options to solve this problematic issue. One possible*

*options is to compare two groups – one group that will be allowed to blink, and another group*

*that won’t be able to blink (by using dedicated eye drops). Another possible option is by*

*comparison between trials / time windows with blinks and those without blinks. If the authors*

*find meaningful differences between the groups, the interpretation of the original results will be*

*more reliable. Without this comparison it is difficult to understand the weight of this possible*

*artifact in pupillometry studies.”*

**Our reply to [C2-1]**

We agree with the reviewer that the weight of BPR in pupillometry would be better appreciated if there is a more straightforward demonstration of the effects of BPR correction. We thank the reviewer for suggesting two possible options for making such a demonstration. We reasoned that the second option, i.e., comparing the ‘blink-affected’ trials and the ‘blink-free’ trials, is more suitable than the first option, i.e., comparing the ‘blink-allowed’ condition and the ‘blink-suppression’ condition. Of course, the first option requires additional data collection. However, a more important reason for preferring the second option to the first one is our concern that active suppression of blinks is likely to distort the genuine time course of pupil size (see a new section of DISCUSSION in the revised manuscript (“Practical considerations for avoiding BPR-related problems”, which was added as our reply to one of the Reviewer #1’s comments (**[C1-4]**)). So, we carried out the second analysis on the auditory oddball detection data (wherein individual trials are brief (2 s) and thus ideal for trial-to-trial analysis) and summarized the results with a new paragraph (the fourth paragraph under the RESULTS section titled “Correcting the auditory oddball data for BPR”) and a new figure (Figure 6) in the revised manuscript.

We note that the reviewer, while raising a possibility that the results shown in Figure 5 might not indicate the association (or causal relationship) between blinks and BPR, pointed out the wavy modulation of pupil size in the BPR-corrected time course of pupil size (“*For example, in*

*Fig. 5C it seems that there are dips after about 5 seconds and 20 seconds. These dips looks pretty*

*much the same as other dips that were associated by the authors to eye blinks and therefore to*

*BPR. Moreover, it seems that there are oscillations in pupil size that cause dipping about every 5*

*seconds (0.2 Hz).”*). This wavy time course was referred to again in one of the minor points of the reviewer (see **[M2-5]** in the below: *“5. Lines 603-609: Why were the fixation colors different between trials? With respect to the first major point, maybe these contrasts were the reason for the 0.2Hz oscillations.”*). We were quite perplexed with this comment and cautiously guess that the reviewer was confused about the time scale at which the time course of pupil size is described: the time unit used in all the plots of Figure 5 was “second” and thus the wavy modulation occurs not at 0.2 Hz but at 2 Hz. If we misunderstood the reviewer’s point, please let us know. However, more importantly, the reviewer’s pointing out this wavy modulation (and associated possible suspicion that comes with it) made us think that it’s better to offer some explanation for the wavy curve (we thank the reviewer for that). Although not 100% sure, we think that the first and second peaks are likely to reflect the previously known pupil responses that are associated with a sound stimulus and a manual response to it, given that subjects heard a sound and judged the tone of the sound by pressing one of the two assigned keys each and every trial. This conjecture is in line with the results that the blink-free trials (the gray lines in Figure 6A in the revised manuscript) and the corrected blink-affected trials (the green line in Figure 6A) both exhibited the double-peak pattern at 2 Hz. And we looked up the literature on pupillometry experiments with the auditory oddball task and found that the wavy pattern has been reported in the studies where subjects pressed a button on each trial like in our study [1,2]. In the revised manuscript, we offered our interpretation for this wavy modulation at 2 Hz in the fourth paragraph of the RESULTS section titled “Correcting the auditory oddball data for BPR”.

**[C2-2]** Reviewer #2’s comment on showing the contribution of the BPR correction in the cognitive experiments

*“2. In addition to the (relatively) steady state measurement of the pupils (the fixation task), the*

*authors ran more two independent cognitive tasks. While the results of the fixation task were*

*relatively replicated in these tasks, the main idea behind the possible artifact is still not clear. Yes,*

*indeed, the mean was pretty much the same and the scattering was decreased (this makes sense*

*with respect to the first main issue that caused interpolation of a lot of dips). However, the*

*influence of this possible artifact on the actual effects unclear. From the study perspective, the*

*BPR might cause systematic pupil decrement that is not associated with the task (this is a bit of a problematic statement due to the fact that the authors cited studies that associated eye blink rate*

*with task difficulty), therefore, effects should appear (false positive) / disappear (false negative)*

*after the BPR correction. If this is the case here, the authors should present these possible*

*scenarios with / without BPR corrections. Specifically, on one hand, the authors should show*

*effects (in terms of the actual task – e.g., easy vs. difficult) that will appear after correction, and*

*on the other hand, the authors should show effects that will disappear after correction. Without*

*these comparisons, the contribution of the correction is not clear (at least in cognitive tasks).”*

**Our reply to [C2-2]**

As the reviewer pointed out, it would have been a much more straightforward (or more dramatic) demonstration of the effects of BPR correction if we had shown that the effects of interest (i.e., ‘oddity’ and ‘memory load’ in the first and second cognitive experiments, respectively) “*appear (false positive) / disappear (false negative)* *after the BPR correction*”. However, the effects were already statistically significant even before the BPR correction in both of the cognitive experiments. That’s also consistent with what has been previously reported. That’s why we decided to focus on the power analysis and identifying the exact contribution of the BPR correction, which was to decrease the variabilities of pupil-size measurements at the subject-to-subject and blink-to-blink levels (as summarized in Figure 6 and 8 in the previously submitted manuscript). We stress that this specific contribution is highly consistent with the results in the fixation experiment, where we found that BPR substantively varied in both shape and amplitude across individuals and mainly in amplitude across blinks within given individuals. We also stress that what were depicted in Figure 6D and 8E in the old manuscript partly demonstrate what the reviewer wanted to see. That is, there are a certain range of trial numbers (or subject numbers in a realistic situation) wherein the effects of interest disappear (insignificant) before correction and appear (significant) after correction. This was one of the motivations for drawing those plots.

Having explained why we focused on the power analysis, we also understand why the reviewer requested a straightforward demonstration of “false positive” or “false negative”. Although we cannot carry out such a demonstration for the reason pointed out in the above, we thought that it would help readers ‘tangibly sense’ the contribution of the BPR correction if we visually summarize how much the BPR correction increases the effect size in the two cognitive experiments. Thus, in the revised manuscript, we added new figure panels wherein the mean trajectories of pupil size are shown for each level of the main variable and the time courses of t statistics are shown in parallel for the differences between the levels (Figure 7A and 9A) and added several sentences for describing the results (the fifth paragraph of the RESULTS section titled “Correcting the auditory oddball data for BPR” and the second paragraph of the RESULTS section titled “Correcting the delayed orientation estimation data for BPR”).

**[C2-3]** Reviewer #2’s comment on the need for direct comparison of the blink-free and blink-affected trials

*“3. The authors present a model that should solve the BPR artifact. While the model without any*

*doubt changed the general pattern of the pupil size, it is not clear if it was done correctly.*

*Specifically, it is not clear whether the pattern of the pupils after the correction is in line with the*

*expected pattern of the pupils in the case of no eye blinks and moreover, without PBR. Without*

*this important comparison, it is impossible to evaluate the efficiency of the authors’ algorithm.”*

**Our reply to [C2-3]**

We agree with the reviewer on this matter. We addressed this issue as part of our reply to the reviewer’s first major comment (see our reply to **[C2-1]** in the above).

**[C3-1]** Reviewer #3’s comment on the accessibility of the BPR correction toolbox

*“This is an important contribution to cognitive pupillometry methods, especially given that only one previous study has experimented with a modelling approach to correct for the BPR. The convincing demonstration of improved statistical power would however be more exciting if I felt that the BPR modelling approach–described by the authors as 'The proper way of correcting pupillary measurements for BPR'–would be easy to implement on my own data. The author's refer to a 'BPR correction toolbox' throughout the manuscript, but it remains unclear whether this is actually something that is being offered. What does a researcher have to do to apply this method to their own data? If it is overly complicated, I confess that I would probably stick with linear interpolation and incur the penalty to statistical power.”*

*“1. Describe the steps that a researcher must take in order to implement this approach on their own pupil data, and indicate whether a 'BPR correction toolbox' will be made available. If there is not going to be a toolbox and it is time-consuming to implement, this should be listed as a limitation.”*

**Our reply to [C3-1]**

We thank the reviewer for appreciating the methodological contribution of the current manuscript to cognitive pupillometry studies. We also appreciate the reviewer’s constructive suggestions for improving the impact of our findings by increasing the accessibility of our BPR correction method and offering a practical guide for pupillometry researchers. These suggestions are very much in line with those made by the reviewer #1 (**[C1-3]** and **[C1-4]**). So, please read our replies to those comments in the above to see how we addressed this issue in the revised manuscript.

**[C3-2]** Reviewer #3’s comment on a linear interpolation method as a quick and easy alternative solution of BPR correction

*“On the topic of linear interpolation, it may be worth acknowledging that some approaches do attempt to account for the BPR on a blink-by-blink basis. For example, I've previously processed EyeLink data with a toolbox called cili (https://github.com/beOn/cili), which has a function to extend blink end points to the first sample where the z-scored rate of change of the pupil time course drops below 10% of the average within a 100 ms moving window. This does a fairly good job of drawing a line across the BPR and is very easy to implement.”*

*“3. Acknowledge alternatives to dealing with the BPR, such as the linear interpolation approach on a blink-by-blink basis that I mentioned above.”*

**Our reply to [C3-2]**

We thank the reviewer for letting us know about the presence of such a sophisticated toolbox that deals with individual blinks. As the reviewer commented, we learned that this toolbox (called ‘cili’) indeed did a great job in interpolating the blink-affected part of data on a blink-by-blink basis. So, we mentioned this toolbox and briefly described its usefulness in the revised manuscript. However, we stress that this solution is not free from the fundamental limit intrinsic to all kinds of the interpolation-based approach. Of course, the linear interpolation method does a good job of removing BPR. Unfortunately, while doing so, it throws out the signals associated with changes in the cognitive states of researchers’ interest. We repeatedly demonstrated this limit of the interpolation method in our data sets (Figure 6 (old manuscript) / Figure 7 (revised manuscript) for the auditory oddball experiment; Figure 8 (old manuscript) / Figure 9 (revised manuscript) for the delayed orientation estimation experiment). We regret that we should have more explicitly and clearly pointed out this limit of the linear interpolation method. Thus, in the revised manuscript, this limit was more explicitly emphasized in RESULTS sections and discussed in the DISCUSSION section titled “The proper way of correcting pupillary measurements for BPR” (the last three sentences in the first paragraph).

**[C3-3]** Reviewer #3’s comment on the complicated nature of the BPR correction procedure

“The analysis appears to be in good order, but I am not an expert in modelling and therefore do not feel qualified to make comments on the finer details of the analysis. My general feeling is that this may be overcomplicated for the routine analysis of pupillometry data.”

**Our reply to [C3-3]**

We admit that the probabilistic inference algorithm of BPR correction requires an intricate set of computations, which can appear highly complicated to readers without much of modeling experience. To ease this concern, we revised the following in the revised manuscript. First, we reorganized the METHODS and MATERIALS section in which the correction algorithm is formalized (“**The algorithm of correcting pupillary responses for BPR**”). Specifically, we divided the section into two parts, one describing the generative model (a model about how the observed pupil-size measurements are generated) and the other describing the inference model (the model about how the BPR is normatively inferred from the observed pupil-size measurements according to the generative model). In doing so, we clarified what assumptions are introduced on what basis and specified what parameters of the model were actually fit to the data. We expect these revisions to make the proposed algorithm more transparent and accessible to readers without losing scientific rigor. Second, we modified the previous MATLAB toolbox such that any readers, including those without much of modeling experience, can apply the BPR correction algorithm as an out-of-the-box solution to their own data with minimal editing. Finally, we uploaded the toolbox with some documents in which the principle of the BPR correction algorithm is explained along with a demonstration of applying the algorithm to an example data set. Now this package is available for downloading (<https://github.com/yookyung1310/BPR_toolbox>). These guides for applying our algorithm to readers’ own data are briefly described in the revised manuscript both in METHODS and MATERIALS (“**The algorithm of correcting pupillary responses for BPR**”) and DISCUSSION (“**The proper way of correcting pupil-size measurements for BPR**“).

**Minor comments: [M2-12] ~ [M3-7]**

**Reviewer #2’s minor comments:**

**[M2-1]** *“1. Lines 138-142: I’m not entirely sure what is happening here.”*

**Reply:** In the revised manuscript, we rewrote this part in a more accessible way, with a bit more of details, and directed readers to a subsection of METHODS and MATERIALS section for details (“**Analysis of the blink-by-blink variability of BPR in peak amplitude and time-to-peak” under “Data Analysis”**). This subsection was also substantially revised to make the procedure more transparent.

**[M2-2]:** *“2. In most of the figures the legends are missing.”*

**Reply:** In the revised manuscript, we added legends for every symbol, color, and line used in figures.

**[M2-3]:** *“3. Something doesn’t make sense in Fig. 7. After 4 seconds there are a lot blinks (the EBR is*

*relatively large), but the pupil size is still increased (with / without corrections). How it is in line*

*with the main idea of BPR? Why the pupil is sometimes increased and sometimes decreased?”*

**Reply:** First of all, it should be stressed that the pupil-size measurements are not just an outcome of BPR but also influenced by cognitive and spontaneous states, as we assumed in our generative model (as depicted in Figure 4A). We interpret that the pupil size increases after 4 seconds despite of the increase of EBR because the pupil size tends to increase when subjects are required to (‘cognitively’) retrieve the memory of the target orientation and make manual adjustments for estimation report. Both of these processes are known to increase the pupil size (Hong et al., 2014; Murphy et al., 2011; Robison and Unsworth, 2019). Of course, blinks will decrease the pupil size, but blinks do not occur not that often (~0.3Hz) and overshadowed by the rapid increase due to the cognitive demand. As demonstrated in Figure 9A, when BPR is corrected with our algorithm, the corrected time course was greater than the uncorrected time course. In the revised manuscript, we explained the rapid rise during the estimation epoch of the delayed orientation estimation task (the second paragraph of “Correcting the delayed orientation estimation data for BPR”).

**[M2-4]** *“4. Lines 569-574: Was the exclusion (of both trials and subjects) also based on task performance? If not, why?”*

**Reply:** We did not exclude trials or subjects based on task performance. Task performance was generally good for all the participants, and we could not find any particular reasons to exclude based on task performance. Instead, we excluded the entire data sets from a few subjects when the recording quality was poor, when subjects dozed off during experiments, or when the blink rate was unusually high. We revised the “Subjects” section of METHODS and MATERIALS and stated that how many subjects are discarded from further data analysis on what basis.

**[M2-5]** *“5. Lines 603-609: Why were the fixation colors different between trials? With respect to the first major point, maybe these contrasts were the reason for the 0.2Hz oscillations.”*

**Reply:** The color of the fixation cross was not changed at all over trials within individual runs. The color of the fixation mark was fixed at a specific value such that the visibility was secured against the background luminance. To avoid confusion, we revised the “Stimuli and procedure” section of METHODS and MATERIALS.

**[M2-6]** *“6. Line 653: How did the authors separate between eye-blinks and other artefacts (e.g., noise / head movements).”*

**Reply:** First of all, as we stated in the “Stimuli and procedure” section of METHODS and MATERIALS, we used a chinrest to avoid head movements and to keep the distance between the eyes and the eye-tracker. Thus, the outputs from the eye-tracker were not that noisy and blinks were readily discriminated from other miscellaneous background noises.

**[M2-7]** *“7. Lines 653-656: Please note that this approach is less efficient compared to the noise-based*

*approach (Hershman et al., 2018).”*

**Reply:** Yes. We thank the reviewer for letting us know about this nice work. We acknowledged and referred to Hershman et al. (2018) in the revised manuscript (“Removal of artifacts around single blink events” in METHODS and MATERIALS).

**[M2-8]** *“8. Line 659 (“Blink onsets and offsets were defined from the start and end points of the blinks”): This sentence is not clear. How were the onset and the offset defined?”*

**Reply:** We regret that we did a poor job describing the procedure of defining the starting and end points of a blink event. In the “The definition of single blink events” subsection in MATERIALS and METHODS, we detailed the procedure.

**[M2-9]** *“9. Lines 663-665: A period of 150 ms is problematic. Please see Hershman et al.’s (2018) discussion about it.”*

**Reply:** We thank the reviewer for the heads up. We learned the importance of this procedure, not in DISCUSSION but in INTRODUCTION of Hershman et al.’s (2018) though (please correct us if we misunderstood the point). We admit that our procedure, which is being adopted conventionally, may throw away the valuable information that exists right before blinks. However, while acknowledging the potential problem of our procedure, we do not think that its impacts on the BPR correction is negligible because the time window of BPR confounds (0.5~ 3 seconds posterior (not prior) to blinks) is not overlapped with the short period prior to blinks.

**[M2-10]** *“10. Lines 667-672: This part is not clear. Why was smoothing required?”*

**Reply:** We regret that we should’ve explained why we carried out smoothing. In the “Removal of artifacts around single blink events” subsection in Materials and Methods, we explained the reason for the smoothing procedure.

**[M2-11]** *“11. Lines 773-776: Why were these time windows chosen?”*

**Reply:** In the “Analysis of blink rate and pupil data in the auditory odd-ball and delayed orientation estimation experiments” subsection of MATERIALS and METHODS, we explained why the specific time windows were chosen. The time window of delayed orientation estimation task was changed, because the previous window was set somewhat arbitrary. Since we wanted to define a time window that captures the memory retrieval effects most effectively, we defined it as the time points where the pupil-size measurement was above 75% of the maximum of the grand average across the memory load conditions. As a result, the time window became narrower (1.22-2.38 seconds) than before (0.5-3 seconds) (See ‘Analysis of blink rate and pupil data in the auditory oddball and delayed orientation estimation experiments’).

**[M2-12]** *“12. The BPR correction algorithm (lines 722-768): The algorithm is pretty difficult to follow.*

*Simplification of this section is required. In addition, did the authors share the source code? Free*

*downloading of the Matlab / Python / R code would be useful here (both for the readers and for*

*the reviewers)”*

**Reply:** We thank the reviewer for the suggestions. Please see our replies to the other reviewers’ comments (**[C1-3]** and **[C3-3]**), where we stated how we addressed these points in the revised manuscript.

**Reviewer #3’s minor comments:**

**[M3-1]** *“1. With EyeLink systems, optical distortion of the pupil image (caused by changes in gaze position) affects the pupil data by up to 10%. This should be acknowledged, and ideally a simple analysis provided showing that participants performed the fixation task adequately. It would also be helpful to indicate whether gaze related trial exclusion criteria were applied for the fixation and oddball tasks.”*

**Reply**: We thank the reviewer for pointing out this important methodological issue. In the “Stimuli and procedure” subsection of MATERIALS and METHODS of the revised manuscript, we explicitly stated “Gaze related trial exclusion was not applied”. By the way, we measured how much gaze deviated from the fixation target and found that the amount of deviance was not severe (1.45 ± 0.09, 1.79 ± 0.94, 1.84 ± 0.64 deg (mean ± SD across subjects for the fixation, auditory oddball, and delayed orientation estimation experiments, respectively), and 0.92 ± 0.43, 1.01 ± 0.52, 0.94 ± 0.38 deg (mean and ± SD of within-subject SDs for the three experiments, respectively). While we acknowledge that there might existed some noise from these gaze deviances, the amount of noise would be not large in normalized pupil scale (our eye-tracker setting is similar with pupil diameter=4.76mm, far layout condition of Hayes & Petrov 2016.) [3]. Although it is not large, we share the reviewer’s concern and thus checked the possibility that the pupil change confounded by gaze deviance affected our claims about the effect of blinks on pupil-size measurements. We reasoned that our claim is threatened only when blinks are correlated, either positively or negatively, with the amount of gaze deviances. We found that the cross-correlation between blink events and gaze deviances were close to zero or almost negligible (maximum cross correlation < 0.025 in any runs, in both horizontal and vertical gaze deviances). In the revised manuscript, we summarized these results in a new subsection of MATERIALS and METHODS (“Analyses on gaze deviance and potential dependency between blink and gaze”, lines 802 ~ 816).

**[M3-2]** *“2. Please clarify whether any of the participants took part in more than one of the three studies outlined in the manuscript.”*

**Reply:** In the “Subjects” section of Materials and Methods of the revised manuscript, we clearly stated this information (“A total of 13 subjects participated in more than one experiment: 11 in all of the three experiments; 2 in the fixation and auditory odd-ball experiments”).

**[M3-3]** *“3. Please indicate whether a 'BPR toolbox' will be made available, and if so, where it is maintained and and how to use it.”*

**Reply:** Please see our replies to **[C1-3]** and **[C3-3]** in the above to see how we addressed this issue in the revised manuscript.

**[M3-4]** *“4. Corneal flux density (c) is included as a parameter in the model. Does this mean it is necessary for researchers to obtain their own measurements of corneal flux density in order to accurately model the BPR?”*

**Reply:** We feel sorry for making the reviewer confused about the role of the variable c. Actually, this variable should have been just “the lighting condition.” In a nutshell, the variable c plays the same role as the variable s (subject), meaning that the model should be applied to separately to different individuals and to different lighting conditions. In the revised manuscript, we changed the name of variable and clarified the meaning of these two “categorical (nominal)” variables (the second and third paragraphs of the “The algorithm of correcting pupillary responses for BPR” in MATERIAL and METHODS).

**[M3-5]** *“1. The meaning of the small lines on the x-axis in Figure 2 should be clarified in the figure caption. I assume they are indicating the time-to-peak for the respective traces?*”

**Reply:** Yes, they are. We revised the figure caption of figure 2 for this.

**[M3-6]** *“2. The readability of the figures could be improved by increasing the DPI and possibly font size.”*

**Reply:** The font of figures may be little bit small. DPI was 300 pixels/inch, which is required for PLOS One format. We guess that a merged PDF file that was provided for the reviewers included figures with poor resolution. We increased font size and added more legends to help readers understand figures clearly.

**[M3-7]** *“The manuscript is presented in an intelligible fashion but lapses frequently in grammar and phrasing, sometimes tone. With this in mind, I recommend additional proof-reading and copy editing. Special attention should be given to the abstract, figure captions and the Materials and Methods Section.”*

*“Example minor points:*

*1. Line 45 - BPR is defined here as blink-locked pupillary response, but was defined as as blink-induced pupillary response in the abstract (line 22).*

*2. Line 61 - (about xx in z-score unit) - Estimated value is missing*

*3. Line 563 - 'eyet racker'*

*4. Line 580 - 'Fixaion'*

*5. Line 626 - Review this sentence*

*6. Line 706 - 'we collected acquired'*

*7. Lines 457-460 - review sentence*

*8. Line 481 - 'In average'*

*9. Line 502 - 'vicious confounder' - revise word choice?*

*10. Line 564 - 'corneal reflex' - shouldn't this be 'corneal reflection'?”*

**Reply:** We admit (and regret) that we did not spend sufficient time and effort on making our manuscript readable and sound fluent. In the revised manuscript, we did our best to correct the entire manuscript for grammatical errors and tried to improve its readability and fluency by incorporating the comments and suggestions from writing experts.

References

1.	Hong L, Walz JM, Sajda P. Your eyes give you away: prestimulus changes in pupil diameter correlate with poststimulus task-related EEG dynamics. PLoS ONE. Public Library of Science; 2014;9: e91321. doi:10.1371/journal.pone.0091321

2.	Murphy PR, Robertson IH, Balsters JH, O'connell RG. Pupillometry and P3 index the locus coeruleus-noradrenergic arousal function in humans. Psychophysiology. 2011;48: 1532–1543. doi:10.1111/j.1469-8986.2011.01226.x

3.	Hayes TR, Petrov AA. Mapping and correcting the influence of gaze position on pupil size measurements. Behav Res. 2nd ed. 2015;48: 510–527. doi:10.3758/s13428-015-0588-x
